# Src is essential for the endosomal delivery of the FGFR4 signaling complex in hepatocellular carcinoma

**DOI:** 10.1186/s12967-021-02807-4

**Published:** 2021-04-01

**Authors:** Ji-Yon Shin, Sung-Min Ahn

**Affiliations:** 1 Department of Health Sciences and Technology, GAIHST, Gachon University, Incheon, 21999 Republic of Korea; 2 Department of Genome Medicine and Science, College of Medicine, Gachon University, Incheon, Republic of Korea

**Keywords:** Src, FGFR4, FGF19, EEA1, STAT3, Endosomal delivery, HCC

## Abstract

**Background:**

Hepatocytes usually express fibroblast growth factor receptor 4 (FGFR4), but not its ligand, fibroblast growth factor 19 (FGF19). A subtype of hepatocellular carcinoma (HCC) expresses FGF19, which activates the FGFR4 signaling pathway that induces cell proliferation. FGFR4 inhibitors that target this mechanism are under clinical development for the treatment of HCCs with FGF19 amplification or FGFR4 overexpression. Src plays an essential role in the FGFR1 and FGFR2 signaling pathways. However, it is yet to be understood whether Src has any role in the FGF19-FGFR4 pathway in HCCs. In this study, we aimed to elucidate the role of Src in the FGF19-FGFR4 axis in HCC.

**Methods:**

3 HCC cell lines expressing both FGF19 and FGFR4 were selected. The expression of each protein was suppressed by siRNA treatment, and the activity-regulating relationship between FGFR4 and Src was investigated by westernblot. Co-immunoprecipitation was performed using the FGFR4 antibody to identify the endosomal complex formation and receptor endocytosis. The intracellular migration pathways of the endosomal complex were observed by immuno-fluorescence and nuclear co-immunoprecipitation. Dasatinib and BLU9931 were used for cytotoxicity comparison.

**Results:**

FGFR4 modulates the activity of Src and Src modulates the expression of FGFR4, showing a mutual regulatory relationship. FGFR4 activated by FGF19 formed an endosomal complex with Src and STAT3 and moved to the nucleus. However, when Src was suppressed, the formation of the endosomal complex was not observed. FGFR4 was released from the complex transferred into the nucleus and the binding of Src and STAT3 was maintained. Dasatinib showed cytotoxic results comparable to BLU9931. The results of our study demonstrated that Src is essential for the nuclear transport of STAT3, as it induces the endosomal delivery of FGFR4 in FGF19-expressing HCC cell lines.

**Conclusions:**

We found that Src is essential for the endosomal delivery of the FGFR4 signaling complex in HCC. Our findings provide a scientific rationale for repurposing Src inhibitors for the treatment of HCCs in which the FGFR4 pathway is activated.

**Supplementary Information:**

The online version contains supplementary material available at 10.1186/s12967-021-02807-4.

## Background

Fibroblast growth factor receptor 4 (FGFR4), a receptor tyrosine kinase, is activated by fibroblast growth factor 19 (FGF19) via an endocrine mechanism [1, 2]. The bile acid secreted by the liver after a meal reaches the ileum and stimulates the secretion of FGF19 [3, 4]. The FGF19 secreted into the bloodstream is transported to the liver where it activates the FGF19–FGFR4 axis in hepatocytes, which consequently inhibits the secretion of bile acid and induces the proliferation of hepatocytes [5, 6]. Hepatocytes usually express FGFR4, but not its ligand, FGF19, indicating that the FGF19–FGFR4 axis is tightly regulated in hepatocytes [7, 8].

A certain subtype of hepatocellular carcinoma (HCC) expresses FGF19 [9, 10]. We previously observed that FGF19 is amplified in approximately 5% of the 231 cases of HCC considered in our study [11]. In another study, we analyzed FGF19 amplification in 989 cases of HCC, and reported that the amplification of FGF19 is associated with poor survival and a high risk of recurrence in patients with HCC [12]. Using murine models, Zhou and colleagues demonstrated that FGF19 drives the formation of HCC by phosphorylating Signal Transducer and Transcription 3 (STAT3) [13]. FGFR4 inhibitors are being developed for treating this subtype of HCC, in which the FGF19–FGFR4 axis is activated [14–16]. Hegel and colleagues reported that BLU9931, an FGFR4-specific inhibitor, inhibits the proliferation of cell lines in which the FGF19/FGFR4 signaling pathway is activated, and exhibits antitumor activity as observed in a murine xenograft model carrying the same HCC cell line [17].

Src, a non-receptor tyrosine kinase, participates in signaling essential for cell proliferation, motility and survival [18]. Studies have shown that Src is highly expressed and activated in epithelial cancers such as colon and breast cancer [19, 20]. In 60% of 65 Chinese patients with HCC, Src expression was increased and increased Src expression and activity showed strong correlation [21].

Src plays an essential role in the FGFR signaling pathway [22–24]. Sandilands and colleagues reported that Src plays a crucial role in regulating the FGFR1 signaling dynamics [25]. Li and colleagues reported that FGFR2 and Src have oncogenic synergies, and targeting Src may serve as a therapeutic strategy for prostate cancer, in which the FGFR2-Src axis is active [26]. However, it is yet to be understood whether Src is involved in conjunction with the FGF19–FGFR4 oncogenic pathway.

In this study, we aimed to elucidate the role of Src in the FGF19–FGFR4 axis in HCC. The results of our study demonstrated that Src is essential for the nuclear transport of STAT3, as it induces the endosomal delivery of FGFR4 in FGF19-expressing HCC cell lines.

## Methods

### Cell culture

SNU878 cells were purchased from the Korean Cell Bank, while Hep3B, PLC/PRF/5, and HUH7 cells were procured from ATCC. Hep3B, PLC/PRF/5, HUH7 cells, and SNU878 cells were maintained in RPMI 1640 medium (Thermo Fisher Scientific, 11875-093) supplemented with 10% fetal bovine serum. The cells were incubated in a humidified incubator at 37 °C with 5% CO_2_.

### RNA extraction and real-time PCR

The cells were seeded in 60 mm dishes and allowed to adhere overnight at 37 °C in an atmosphere of 10% CO_2_. The total RNA was extracted using a RNeasy Mini Kit (Qiagen, Valencia, CA), and the complementary DNA (cDNA) was synthesized using a PrimeScript™ 1st strand cDNA Synthesis Kit (Takara, Japan). In order to quantify the transcripts of the genes of interest, real-time PCR was performed using a CFX96 Real-Time PCR system and iQ SYBR Green Supermix (Bio-Rad) for human FGF19 (Forward—5′-GGAGGAAGACTGTGCTTTCG-3′, Reverse—3′- GGCAGGAAATGAGAGAGTGG-5′), human FGFR4 (Forward—5′-CTGCAGAATCTCACCTTGAT-3′, Reverse—3′-TTCTCTACCAGGCAGGTGTA-5′), and human GAPDH (Forward—5′-AGGGCTGCTTTTAACTCTGGT-3′, Reverse—3′-CCCCACTTGATTTTGGAGGGA-5′). The relative mRNA level was calculated using the 2− ΔΔCT method.

### siRNAs and transfection protocol

We purchased pre-designed FGFR4, FGF19, and Src siRNAs, along with the siRNAs in the negative control from Bioneer (Daejeon, South Korea). For transfecting the siRNAs, 3 × 10^5^ cells were seeded in 60 mm culture plates and maintained in culture medium supplemented with 10% FBS at 37 °C in an atmosphere of 5% CO_2_ for 24 h. The cells were transfected with 100 nM siRNA using 40 µL lipofectamine RNAiMAX reagent (Thermo Fisher Scientific) in 300 µL of opti-MEM (ThermoFisher Scientific). The cells were harvested for further analyses after 48 h of transfection.

### Treatment with FGF19 and Src inhibitors

The cells were grown in 60 mm dishes to 80% confluency and maintained in RPMI 1640 medium supplemented with 1% BSA (Sigma-Aldrich, A2058). After 24 h, the cells were treated with 20 µM saracatinib (Selleckchem, Houston, TX, USA) for 24 h. The next day, the cells were treated with 100 ng/ml FGF19 (Rocky. Hill, NJ, USA) for 1 h.

### Cell viability assay

For the cell viability assay, 5 × 10^3^ cells were seeded in 96-well plates and allowed to adhere overnight. The triplicate wells were subsequently treated with dasatinib or BLU9931 (Selleckchem, Houston, TX, USA), serially diluted from 10 µM for 72 h. Cell viability was determined using the Cell Counting kit-8 assay (CCK-8; Dojindo, Japan).

### Western blotting

For protein extraction, the cells directly harvested with 2X lysis buffer (Cell Signaling Technology, 9803), 100X phenylmethanesulfonyl fluoride (PMSF) (Sigma-Aldrich, 78,830), 10X PhosSTOP, and cOmplete Protease Inhibitor cocktail (Roche). The cell lysates (30 µg protein per sample) were separated by electrophoresis, transferred to PVDF membranes (Millipore, IPVH00010), and were incubated with the antibodies. The anti-Src, anti-pSrc, anti-STAT3, anti-pSTAT3, and anti-EEA1 antibodies (1:1000) were purchased from Cell Signaling Technology, while the anti-FGFR4, anti-LaminB1 (1:200), and anti-beta-actin (1:1000) antibodies were purchased from Santa Cruz Biotechnology. The anti-FGF19 (1:500) antibody was procured from R&D Systems, while the anti-phosphotyrosine antibody, clone 4G10 (1:1000) was procured from Millipore. The secondary antibodies used (1:5000) were HRP-conjugated mouse anti-rabbit IgG-HRP, goat anti-mouse IgG-HRP, and bovine-anti goat IgG-HRP-linked antibodies (Santa Cruz Biotechnology). Detection was performed using an ECL™ Prime Western Blot System (GE Healthcare) and an Amersham Imager 600 (GE Healthcare). The bands were quantified with ImageJ, a Java-based image analysis package that is widely used for measuring density.

### Immunoprecipitation

For detection of pFGFR4, cell lysates were incubated with 2 μg of the anti-FGFR4 antibody bound to protein G magnetic beads (Bio-Rad) for 1 h at room temperature. The bound proteins were detected with anti-phosphotyrosine antibody.

### Co-immunoprecipitation (co-IP)

The cells were lysed with 2X lysis buffer, as previously described. Following centrifugation, the whole-cell lysates were incubated with 2 μg of the specified antibody (anti-Src or anti-FGFR4) bound to protein G magnetic beads (Bio-Rad) for 1 h at room temperature. The bound proteins were detected by western blotting.

### Immunofluorescence

The cells were fixed in 4% paraformaldehyde for 10 min. Following permeabilization with 0.3% Triton X-100, the cells were blocked with 5% normal goat serum for 30 min at room temperature. The samples were subsequently incubated with the primary antibodies (anti-pSrc, anti-pSTAT3, 1:250, Cell Signaling Technology, and anti-FGFR4, 1:50, Santa Cruz Biotechnology) for 24 h at 4 °C. After washing, the samples were incubated with the secondary antibodies (goat anti-mouse IgG H&L -Alexa Fluor® 488, goat anti-rabbit IgG H&L-Alexa Fluor®, 594, 1:250, Abcam) for 1 h at room temperature. After three final washes, the cells were mounted with VECTASHIELD Antifade Mounting Medium with DAPI (Vector Laboratories).

### Subcellular fractionation

The cells were washed twice with 1 × PBS, lysed in hypotonic buffer comprising 10 mM Tris–HCl, pH 8.0, 10 mM KCl, 1.5 mM MgCl2, 0.5% Nonidet P-40, 1 mM dithiothreitol, 1 mM PMSF, 10X PhosSTOP, and cOmplete Protease Inhibitor cocktail, and incubated on ice for 15 min. Following incubation, the lysates were centrifuged at 1500×*g* for 5 min. The supernatants were further centrifuged at 16,100×*g* for 20 min, and the pellets were resuspended in nuclear extraction buffer comprising 400 mM NaCl, 1 mM EDTA, 20 mM Tris–Cl, pH 8.0, 1 mM dithiothreitol, 1.5 mM MgCl2, 25% glycerol, 1 mM PMSF, 10X PhosSTOP, and cOmplete Protease Inhibitor cocktail. The resuspended nuclear fractions were centrifuged at 16,100×*g* for 30 min.

### Statistical analyses

All experiments were repeated at least triplicate. GraphPad Prism 5 was used for data analyses. Statistical significance was measured by two-way ANOVA. IC50 values were calculated from a log([drug]) versus normalized response curve fit.

## Results

### FGF19 and FGFR4 expression in HCC cell lines

**Fig. 1:**
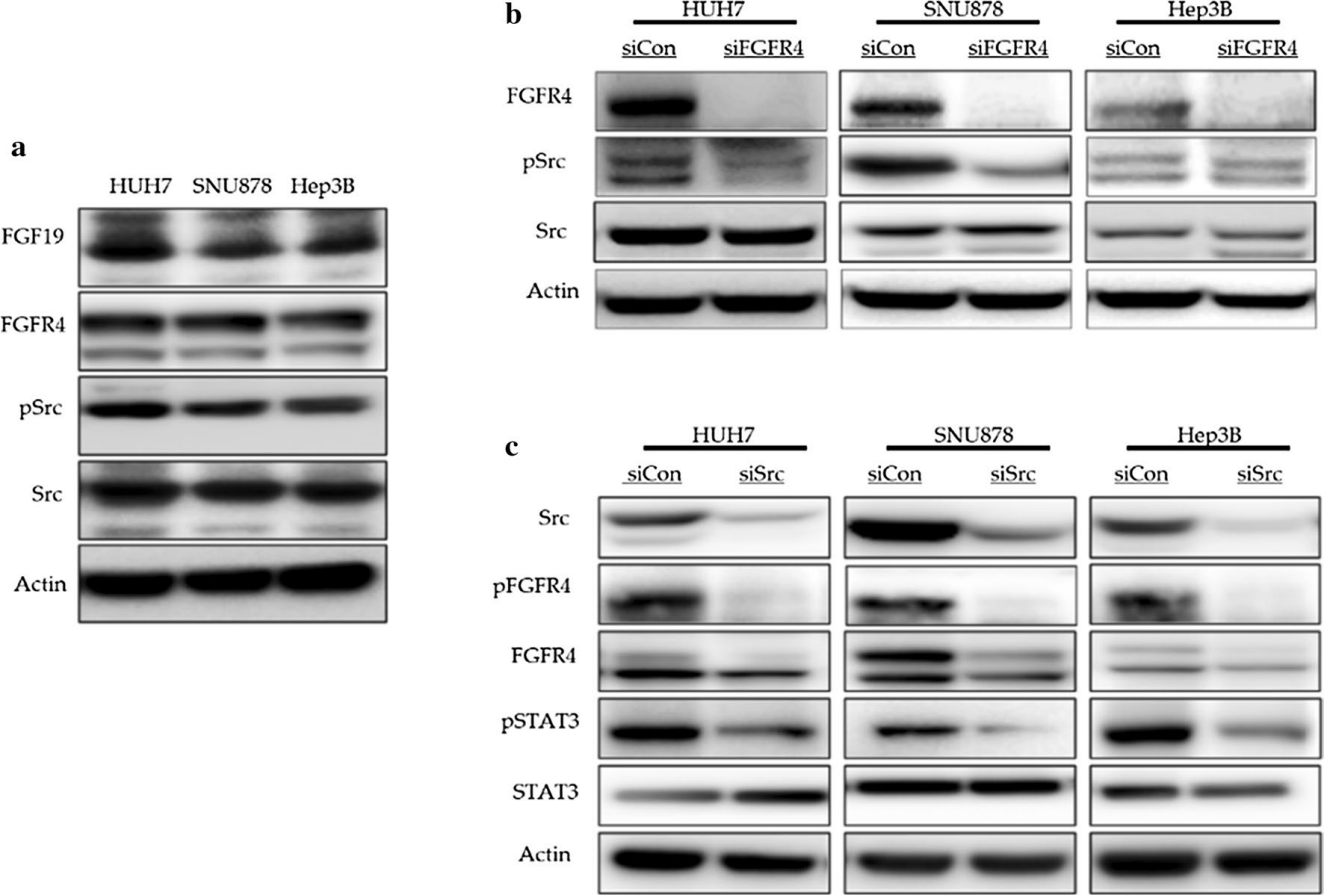
The reciprocal relationship between FGFR4 and Src (**a**) Western blot analysis of FGF19, FGFR4, and Src in the three HCC cell lines. All the cell lines expressed both FGF19 and FGFR4, and Src signaling was intact. **b** Western blot analysis after silencing FGFR4 in the three HCC cell lines. The silencing of FGFR4 decreased Src phosphorylation. **c** Western blot analysis after silencing Src in three HCC cell lines. The silencing of Src decreased the phosphorylation of STAT3 and the expression of FGFR4

In order to investigate the role of Src in the FGF19-FGFR4 signaling axis in HCC, we selected three HCC cell lines that express both FGF19 and FGFR4. We first investigated the transcriptomic profiling data of cancer cell lines in the Cancer Cell Encyclopedia (CCLE) (Additional file 1: Table S1). FGFR4 is expressed in 22 out of 25 HCC cell lines, while FGF19 is expressed in only five HCC cell lines, namely, JHH7, HUH7, HEP3B, SNU878, and SNU761. We then validated the expression levels of FGF19 and FGFR4 proteins and confirmed the expression and phosphorylation of Src in the three HCC cell lines used in this study (Fig. 1a).

### The reciprocal relationship between FGFR4 and Src

FGFR4 was subsequently subjected to siRNA silencing in the three HCC cell lines, which reduced Src phosphorylation. This supported the fact that Src is one of the downstream targets of FGFR4 (Fig. 1b). Following the siRNA silencing of Src in the three HCC cell lines, we observed that Src silencing inhibited the phosphorylation of STAT3, which indicated that Src mediates the signaling between the FGF19–FGFR4 axis and STAT3. We also observed that Src silencing decreased the expression of FGFR4, which indicated that Src, a downstream target of FGFR4, is involved in the transcriptional regulation of FGFR4 (Fig. 1c). These observations were validated in subsequent experiments, which are described hereafter.

### Src is an essential mediator of FGFR4 expression in the FGF19-FGFR4 axis in HCC

**Fig. 2:**
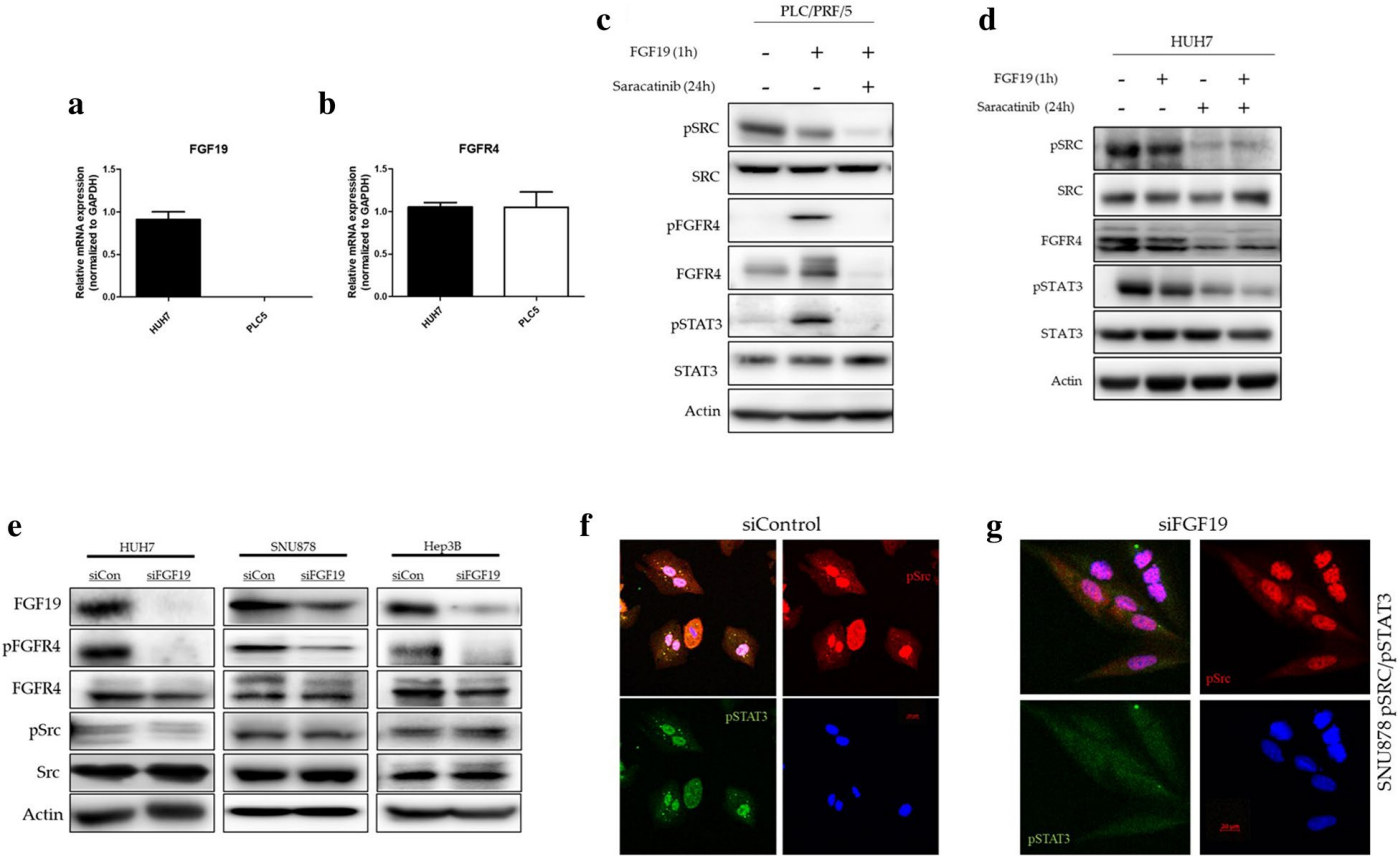
The role of Src in the FGF19-FGFR4 axis in HCC. **a**, **b** The mRNA expression levels of FGF19 and FGFR4 in the PLC5 cell line. The PLC5 cell line expresses FGFR4, but not FGF19. HUH7 was used as a reference sample. **c**, **d** Western blot analysis of the FGF19-FGFR4-Src axis after treating PLC5 and HUH7 cells with the Src inhibitor, saracatinib and recombinant FGF19. Saracatinib treatment decreased the phosphorylation of STAT3 and the expression of FGFR4. **e** Western blot analysis after silencing FGF19 in the three HCC cell lines. The silencing of FGF19 decreased FGFR4 phosphorylation. **f**, **g** Immunofluorescence staining of pSrc (red) and pSTAT3 (green) in SNU878 cells treated with either FGF19 siRNA or control siRNA. FGF19 silencing suppressed pSTAT3, but pSrc was not affected

In order to validate the role of Src in the FGF19–FGFR4 axis in HCC, we used the HUH7 cell line, and the PLC5 (PLC/PRF/5) cell line that expresses FGFR4 but not FGF19 (Fig. 2a, b).

The effects of FGF19 treatment on PLC5 cells are summarized in Fig. 2c. The treatment of PLC5 cells with recombinant FGF19 increased the phosphorylation of FGFR4 and STAT3, the latter being a downstream target of the FGF19–FGFR4 axis. The treatment of PLC5 cells with recombinant FGF19 following treatment with the Src inhibitor, saracatinib, decreased the phosphorylation of STAT3. Treatment with saracatinib substantially reduced the expression levels of FGFR4. All these findings are consistent with the results of the experiments on Src silencing described in b and c of Fig. 1. Adding recombinant FGF19 to HUH7 cells did not increase the level of phosphorylation of Src and STAT3 and expression of FGFR4 (Fig. 2d).

On the other hand, it was observed that the level of pSrc was slightly decreased in PLC5 cells treated with recombinant FGF19, but there was no change in HUH7. We investigated the expression of proteins after inhibiting FGF19 to determine the regulatory relationship between FGF19 and Src for FGFR4 activity. Phosphorylation of FGFR4 and STAT3 was reduced in three HCC cell lines in which FGF19 was inhibited by siRNA. However, the expression level and phosphorylation of Src were not affected by the presence or absence of FGF19 expression (Fig. 2e, f, g). Summarizing these results with Fig. 1c shows that FGFR4 expression by Src and phosphorylation by FGF19 are essential for activation of FGFR4 signaling, and they act independently of each other for FGFR4 action.

### FGFR4 forms an endosomal complex with Src and STAT3

**Fig. 3:**
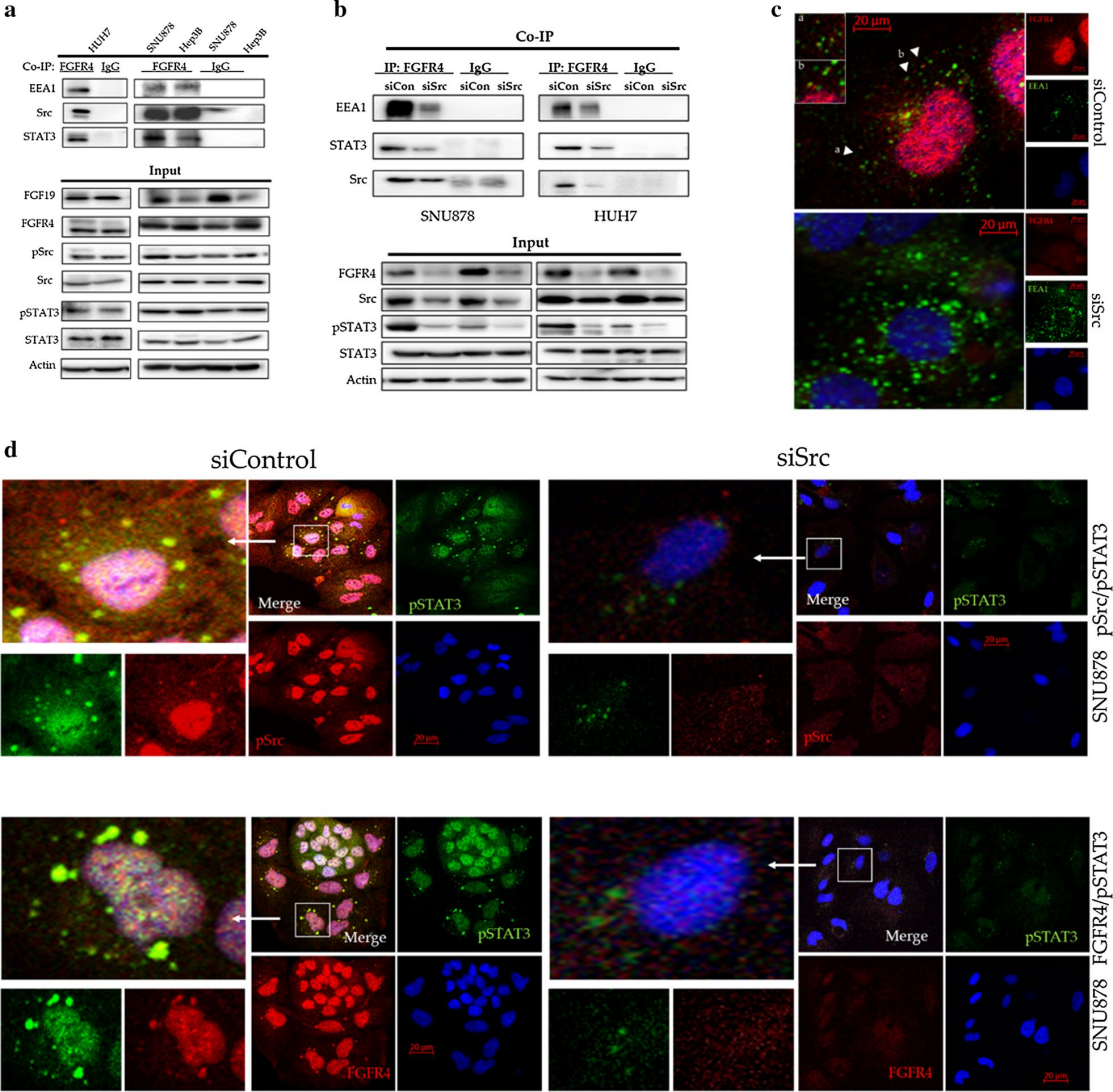
FGFR4 forms an endosomal complex with Src and STAT3, which transfers signals to the nucleus. **a** Western blot analysis of the FGFR4 co-immunoprecipitate. FGFR4 was co-immunoprecipitated with EEA1, Src, and STAT3 in the three HCC cell lines. **b** Western blot analysis of the FGFR4 co-immunoprecipitate following Src silencing. Src silencing substantially decreased the amounts of EEA1 and STAT3 that co-immunoprecipitated with FGFR4. **c** Immunofluorescence staining of FGFR4 and EEA1 in HUH7 cells treated with either Src siRNA or control siRNA. FGFR4 and EEA1 did not co-localize following Src silencing. **d** Immunofluorescence staining of FGFR4 and pSTAT3. FGFR4 and pSTAT3 were co-localized in the cytosol and localized in the nucleus of cells treated with the control siRNAs. The levels of FGFR4 and pSTAT3 were substantially reduced in the cytosol and nucleus of the cells that were treated with Src siRNA. **e** Immunofluorescence staining of pSrc and pSTAT3. It was observed that pSrc and pSTAT3 were co-localized in the cytosol and nucleus of the cells that were treated with the control siRNAs. The levels of pSrc and pSTAT3 were substantially reduced in the cytosol and nucleus of cells treated with Src siRNA

We hypothesized that FGFR4, like FGFR1, may form an endosomal complex that transmits signals to the nucleus. In order to test this hypothesis, we first performed co-IP experiments using an anti-FGFR4 antibody. As demonstrated in Fig. 3a, FGFR4 was found to be associated with EEA1 (Early Endosome Antigen 1), an early endosomal marker, and also with Src and STAT3 in the three HCC cell lines.

In order to investigate the role of Src in the formation of the FGFR4 endosomal complex, we performed co-IP experiments after siRNA silencing of Src in SNU878 and HUH7 cell lines. As demonstrated in Fig. 3b, Src silencing substantially decreased the amount of EEA1 and STAT3 that co-precipitated with FGFR4.

This finding was further validated by immunofluorescence staining of HUH7 and SNU878 cells. It was observed that FGFR4 co-localized with EEA1 in the cytosol of cells that were treated with the control siRNA. However, FGFR4 and EEA1 did not co-localize in the cells that were treated with Src siRNA (Fig. 3c). FGFR4 and pSTAT3 co-localized in the cytosol and localized in the nucleus of cells that were treated with the control siRNAs. The levels of FGFR4 and pSTAT3 were substantially reduced in the cytosol and nucleus of cells treated with Src siRNA (Fig. 3d). We observed that pSrc and pSTAT3 were co-localized in the cytosol and nucleus of cells that were treated with the control siRNAs. The levels of pSTAT3 were substantially reduced in the cytosol and nucleus of cells that were treated with Src siRNA (Fig. 3e). Altogether, these findings indicated that Src is essential for the dynamic activity of FGFR4 in the formation of an endosomal complex with EEA1, pSrc, and pSTAT3, which are subsequently delivered to the nucleus. While FGFR4, pSrc, and pSTAT3 are transported to the nucleus, EEA1 is not.

### Fate of the FGFR4 signaling complex in the nucleus

**Fig. 4:**
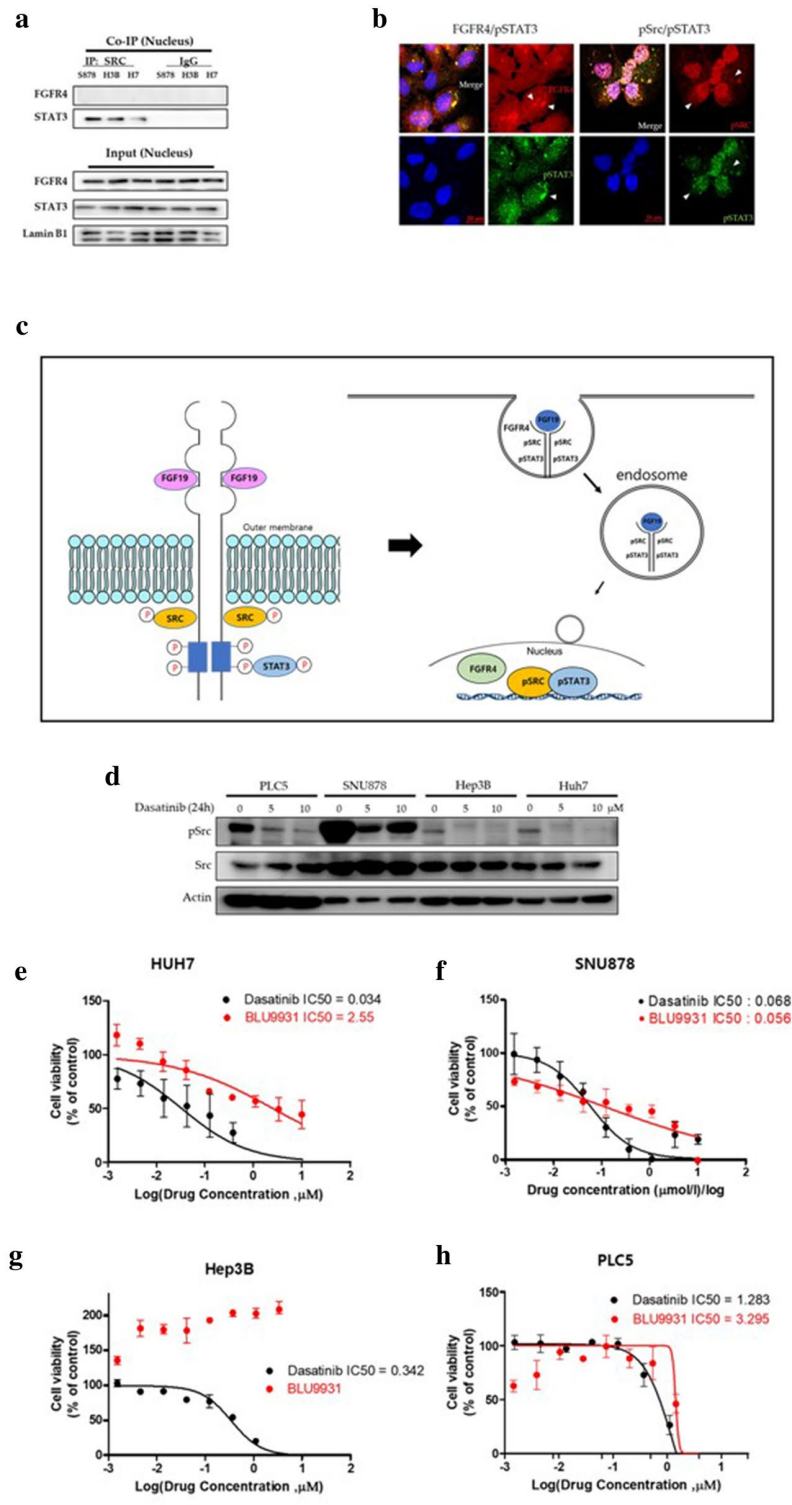
Co-localization of signaling molecules in the nucleus. **a** Western blot analysis of the anti-Src antibody co-immunoprecipitate in the nuclear fractions of the three HCC cell lines. Src co-immunoprecipitated with STAT3, but not with FGFR4. **b** Immunofluorescence staining of FGFR4, pSrc, and pSTAT3 in SNU878 cells. FGFR4 and pSTAT3 were co-localized in the cytosol, but not in the nucleus. pSrc and pSTAT3 were co-localized in the cytosol and nucleus. **c** Schematic model depicting the endosomal delivery of the FGFR4 signaling complex. FGF19 activates FGFR4, which forms an endosomal complex with pSTAT3 and pSrc, and is transported to the nucleus. **d** Effects of dasatinib on Src phosphorylation. The level of Src phosphorylation decreased with dasatinib treatment. **e**–**h** Viability of HCC cells treated with the Src inhibitor, dasatinib, or with the FGFR4 inhibitor BLU9931 (**P < 0.0001). The cytotoxic efficacy of dasatinib was comparable to that of BLU9931

The results of our study demonstrated that FGFR4 forms an endosomal complex with Src and STAT3, which are delivered to the nucleus. We subsequently proceeded to investigate the fate of the endosomal complex in the nucleus. To this end, we performed anti-Src co-IP experiments using nuclear extracts of three HCC cell lines, SNU878 (S878), Hep3B (H3B), and HUH7 (H7). As summarized in Fig. 4a, Src co-immunoprecipitated with STAT3, but not with FGFR4, in the nuclear extracts of the three HCC cell lines.

This finding was further validated by immunofluorescence staining of SNU878 cells. As demonstrated in Fig. 4b, FGFR4 and pSTAT3 were co-localized in the cytosol, but not in the nucleus. pSrc and pSTAT3 were co-localized in the nucleus as well as in the cytosol. Based on these findings, we conceptualized a model of the endosomal delivery of the FGFR4 signaling complex (Fig. 4c). In this model, FGF19 activates FGFR4, which forms an endosomal complex with pSTAT3 and pSrc, which is transported to the nucleus. Within the nucleus, FGFR4 dissociates from the complex, while pSrc and pSTAT3 remain bound to the complex for transcriptional regulation. As Src is essential for the endosomal delivery of the FGFR4 signaling complex, we hypothesized that an Src inhibitor may inhibit the proliferation of the HCC cell lines in which the FGF19–FGFR4 axis is active. To test this hypothesis, we compared the cytotoxic effects by Src inhibition. The Src inhibitor dasatinib reduced Src phosphorylation in HCC cell lines (Fig. 4d). We compared the efficacy of the dasatinib with the FGFR4 inhibitor BLU9931 in 3 HCC cells and in PLC5 cells, an FGF19 negative control. As summarized in Fig. 4e–h, the in vitro cytotoxicity of dasatinib was superior, or at least comparable, to that of BLU9931.

## Discussion

In this study, we aimed to elucidate the role of Src in the FGFR4 signaling pathway in HCC. The results of our study demonstrated that Src is essential for the endosomal delivery of the FGFR4 signaling complex in HCC. To our knowledge, this study is the first to demonstrate the role of Src in the FGFR4 signaling pathway in HCC.

Clinically, our finding opens a novel avenue of treatment for HCC, which is based on the activation of the FGFR4 pathway by Src inhibitors. FGFR4 inhibitors are under clinical development for the treatment of HCCs with FGF19 amplification or FGFR4 overexpression [27, 28]. Src inhibitors such as dasatinib and bosutinib have been approved for other malignancies [29–31]. Among these two drugs, we obtained significant results in the cell viability test using dasatinib. Our findings provide a scientific rationale for repurposing Src inhibitors for the treatment of HCCs in which the FGFR4 pathway is activated. However, except for SNU878, the cytotoxic effect of BLU9931 was not significant, and in particular, Hep3B slightly increased cell growth. In 2020, Seitz and colleagues identified the role of FGF9 in the HSC-HCC crosstalk. According to the study, Hep3B expressing FGF9 mRNA was resistant to BLU9931 treatment, which reconciles with our data from Hep3B [32].

We showed through Fig. 2 that Src induces the expression of FGFR4 and that FGF19 activates the expressed FGFR4, so that each is tightly regulated by division of roles. These results suggest that Src activity should precede the FGF19-FGFR4 axis. In addition, in Fig. 4g, the increased sensitivity of PLC5 to dasatinib, in comparison to BLU9931, may be explained by its broader target specificity. Unlike BLU9931, a type I kinase inhibitor highly specific for FGFR4, dasatinib is a type II kinase inhibitor with multiple targets such as Src, Abl, c-Kit, and ephrin receptors [33]. Therefore, it may have cytotoxic effects, not through FGF19-FGFR4-Src.

One of the limitations of this study is that the anti-pFGFR4 antibody is unavailable for immunocytochemical studies. Theoretically, the use of anti-pFGFR4 antibodies is considered to be ideal in immunocytochemistry studies, due to the fact that pFGFR4, pSrc, and pSTAT3 form the signaling complex. For the immunocytochemistry experiments, we used anti-pSrc and anti-pSTAT3 antibodies for confirming that pSrc and pSTAT3 were transferred to the nucleus and remained bound together. However, we used an anti-FGFR4 antibody, as the anti-pFGFR4 antibody, which is more suitable for immunocytochemistry studies, was unavailable. Anti-FGFR4 antibodies have been used instead of anti-pFGFR4 antibodies for immunofluorescence staining in similar studies [34–36].

In this study, it was found that Src not only increases the expression of FGFR4, but also uses FGFR4 as a shuttle to move to the nucleus with STAT3. In addition, in the nucleus, Src maintained binding to STAT3, which means that Src may be involved in the transcriptional regulation of STAT3.

In 2018, Huang and colleagues reported that Src activates STAT3 and forms an Src-STAT3 enhanceosome in the nucleus for inducing gene regulation and the proliferation of breast cancer cells [37]. In future, we intend to perform a more thorough investigation of the transcriptional regulation of the Src-STAT3 enhanceosome in HCCs in which the FGFR4 pathway is activated.

## Conclusions

In conclusion, we found that Src is essential for the endosomal delivery of the FGFR4 signaling complex in HCC. Our findings provide a scientific rationale for repurposing Src inhibitors for the treatment of HCCs in which the FGFR4 pathway is activated.

## Supplementary Information

**Additional file 1: Table S1.** CCLE data of HCC cell lines in ascending order by FGF19 expression.

## Data Availability

All data are available in the manuscript or upon request to the authors.

## Abbreviations

FGF: Fibroblast growth factor
FGFR: Fibroblast growth factor receptor
HCC: Hepatocellular carcinoma
STAT3: Signal Transducer and Transcription 3
EEA1: Early Endosome Antigen 1
