# Supplementary material for: Src is essential for the endosomal delivery of the FGFR4 signaling complex in hepatocellular carcinoma
Source: J Transl Med. 2021 Apr 1;19:138. doi: 10.1186/s12967-021-02807-4 (PMC8017611; doi:10.1186/s12967-021-02807-4)
Supplement: Supplementary file 1 — Additional file 1: Table S1. CCLE data of HCC cell lines in ascending order by FGF19 expression. [file 12967_2021_2807_MOESM1_ESM.pdf]

Supplementary Materials: Src is essential for the endosomal delivery of the FGFR4 signaling complex in hepatocellular carcinoma

Jiyon Shin, Sung-Min Ahn

Gene	FGF19	FGFR4
JHH7_LIVER	5.809659417	6.597133935
HUH7_LIVER	4.675470603	7.146082757
HEP3B217_LIVER	3.967053217	6.947726818
SNU878_LIVER	3.811325691	5.235653603
SNU761_LIVER	1.726756162	5.616695557
HUH1_LIVER	0.661743419	4.516588416
JHH5_LIVER	0.466919602	5.884089637
NCIH684_LIVER	-0.062848468	6.615184747
SNU475_LIVER	-0.429190005	1.471142693
SNU886_LIVER	-2.277027727	4.003881067
HUH6_LIVER	-2.928518324	6.102924655
LI7_LIVER	-3.221684299	5.691462929
HEPG2_LIVER	-3.794127719	6.792763689
JHH1_LIVER	-4.17409277	5.233902391
SNU182_LIVER	-4.584233994	1.151388073
PLCPRF5_LIVER	-4.691174665	6.118252146
SNU398_LIVER	-4.885274622	3.92073683
HLF_LIVER	-5.63978866	1.210866464
JHH6_LIVER	-5.700661163	-1.673435693
SNU387_LIVER	-5.989040006	-4.634239714
SNU449_LIVER	-6.257955113	5.519566911
JHH2_LIVER	-7.079038611	2.540204205
SKHEP1_LIVER	-7.153068002	3.47027421
JHH4_LIVER	-13	5.540597038
SNU423_LIVER	-13	-1.214111962
ALEXANDERCELLS_LIVER		
C3A_LIVER		
HLE_LIVER		

Table. S1 CCLE data of HCC cell lines in ascending order by FGF19 expression. HCC cell lines showing FGF19 and FGFR4 mRNA overexpression are JHH7, HUH7, SNU878, HEP3B, and SNU761. FGFR4 mRNA overexpression was observed in all cell lines except 3 of the analyzed samples.
